# Generation of highly amenable cellulose-Iβ via selective delignification of rice straw using a reusable cyclic ether-assisted deep eutectic solvent system

**DOI:** 10.1038/s41598-020-80719-x

**Published:** 2021-01-15

**Authors:** Chiranjeevi Thulluri, Ravi Balasubramaniam, Harshad Ravindra Velankar

**Affiliations:** Bioprocess Division, Hindustan Petroleum Corporation Limited, HP Green R&D Centre, KIADB Industrial Area, Tarabahalli, Devanagundi, Hoskote, Bangalore, 560067 India

**Keywords:** Green chemistry, Biofuels, Renewable energy

## Abstract

Cellulolytic enzymes can readily access the cellulosic component of lignocellulosic biomass after the removal of lignin during biomass pretreatment. The enzymatic hydrolysis of cellulose is necessary for generating monomeric sugars, which are then fermented into ethanol. In our study, a combination of a deep eutectic (DE) mixture (of 2-aminoethanol and tetra-*n*-butyl ammonium bromide) and a cyclic ether (tetrahydrofuran) was used for selective delignification of rice straw (RS) under mild conditions (100 °C). Pretreatment with DE-THF solvent system caused ~ 46% delignification whereas cellulose (~ 91%) and hemicellulose (~ 67%) recoveries remained higher. The new solvent system could be reused upto 10 subsequent cycles with the same effectivity. Interestingly, the DE-THF pretreated cellulose showed remarkable enzymatic hydrolysability, despite an increase in its crystallinity to 72.3%. Contrary to conventional pretreatments, we report for the first time that the enzymatic hydrolysis of pretreated cellulose is enhanced by the removal of lignin during DE-THF pretreatment, notwithstanding an increase in its crystallinity. The current study paves way for the development of newer strategies for biomass depolymerization with DES based solvents.

## Introduction

The primary objective of all biomass conversion processes is to overcome the barriers of recalcitrance posed by the biomass in a cost-effective and an efficient manner^1^. The polymeric nature of lignin, cellulose and hemicelluloses and their typical arrangements in lignocellulosic materials impart structural integrity to biomass^2^ due to which, energy intensive techniques (pretreatment, enzymatic hydrolysis, fermentation) are required to depolymerize and convert the cellulosic carbohydrates into ethanol^3,4^.

Both, bacteria and fungi produce cellulases. The protein expression levels in fungi are higher than in bacteria and therefore fungi are extensively used for large-scale production of cellulases^5^. Nevertheless, problems encountered during fungal fermentations namely mass transfer limitations, longer incubation periods etc., have resulted in lower enzyme production, thereby increasing their cost of production^6^, due to which 2nd generation ethanol technologies are still not commercially viable^4^. Related reports suggest that bioprocess optimization efforts have resulted in improved cellulase secretion and productivities, but with limited success. It seems, the way forward for enzymes is to enhance their titers using genetically engineered fungi, or to reduce their dosages by efficient recycling^7^ or by improving the amenability of pretreated cellulose during pretreatment.

Pretreated cellulose generated by most of the thermochemical pretreatments (using acid, alkali, steam, ammonia etc.) requires around 20 filter paper units per gram of dry solids to achieve near-complete hydrolysis^8–11^. Some of the earlier reports indicate that cellulase requirement can be lowered below 20 FPUg^−1^ upon pretreatment with catalysts such as hydrogen peroxide-assisted sodium carbonate, mild acid-catalyzed atmospheric glycerol (organosolv) or potassium hydroxide^12–14^. In particular, pretreatment of biomass with dilute acids, ionic liquids, via steam explosion or the hydrothermal process reportedly increased the porosity of lignocellulosic substrates, due to removal of hemicelluloses and lignin resulting in rapid hydrolysis due to enhanced access of cellulases to cellulose^15–19^. Although ionic liquids are very effective pretreatment catalysts, these are expensive to produce, are non-biodegradable and toxic to biocatalysts^20,21^. Recently, a new class of catalysts called as the deep eutectic solvents (DESs) that consist of a eutectic-mix of Lewis or Brønsted acids and bases with atleast one hydrogen bond donor (HBD) and an acceptor (HBA) were found to be equally promising for delignification^22,23^. Moreover, DESs are effective, cheaper, biodegradable and recyclable solvents^24^; albeit being slow-acting (12–24 h)^25,26^ and may show some deterioration over time^27,28^. Overall, the choice of the pretreatment influences the nature of cellulose-rich solids and its amenability to hydrolysis by cellulases.

The rigidity or incalcitrance of cellulose is usually attributed to the presence of a large number of inter- and intra-molecular hydrogen bonds associated with hydroxyl moieties located in the glycosidic rings^29^. Four different allomorphs (I, II, III and IV) of cellulose have been identified^30^. Cellulose I is the most abundant form and it occurs widely in plant cell walls while cellulose II is observed in mercerized (alkali-treated) cotton^31^. Cellulose III is generated by treatment of Cellulose I (native) with anhydrous ethylamine or liquid ammonia whereas cellulose IV, which is usually associated with the production of high-performance rayon, is produced by thermo-chemical (glycerol) treatment of Cellulose II^31^. Cellulose I further exists as two sub-polymorphs (i.e. Iα and Iβ) wherein Iα is mainly found in algae and bacteria while Iβ coexists with 1α in higher plants. The Iα form can be irreversibly converted into its thermodynamically stable 1β form under different conditions^32^. The crystallinity of cellulose depends upon the ratio of cellulose I and amorphous regions present in the biomass. Further, glucan chain orientations are differentiated into five lattice planes (1–10, 110, 004, 021, 200) as per the intensities of inter and intra hydrogen bonding (200 > 021 > 004 > 110 > 1–10) due to which, their hydrolysability by cellulases (200 < 021 < 004 < 110 < 1–10) is affected^33,34^. Usually, increased hydrogen bonding in cellulose reduces its amenability to cellulases and vice versa^35^. During the enzymatic hydrolysis of cellulose, the amorphous portion gets hydrolyzed faster than the crystalline region and as the reaction progresses, the porosity of biomass (caused by lignin dislocation and removal of hemicellulose) increases, resulting in higher enzyme infiltration and hydrolysis^15^. Most of the thermochemical pretreatments aim to alter the recalcitrant cellulose structure into its amorphous form before hydrolyzing it into sugars. As described above, the recalcitrant form of cellulose mainly comprises of cellulose I allomorph with dominant lattice planes 200 or 021. An observation emerging out of a study by Hall et al. indicated that lattice plane 021 was more accessible to cellulases than 200^36,37^ which provides a definite direction for the development of future pretreatments.

In the present work, we report the development of a new DE-THF solvent system for carrying out delignification of rice straw, effectively for upto ten reuses. The resultant cellulose rich solids are crystalline but easily amenable to even lower dosages of cellulases.

## Results and discussion

### Effect of pretreatment on chemical composition of biomass

**Figure 1:**
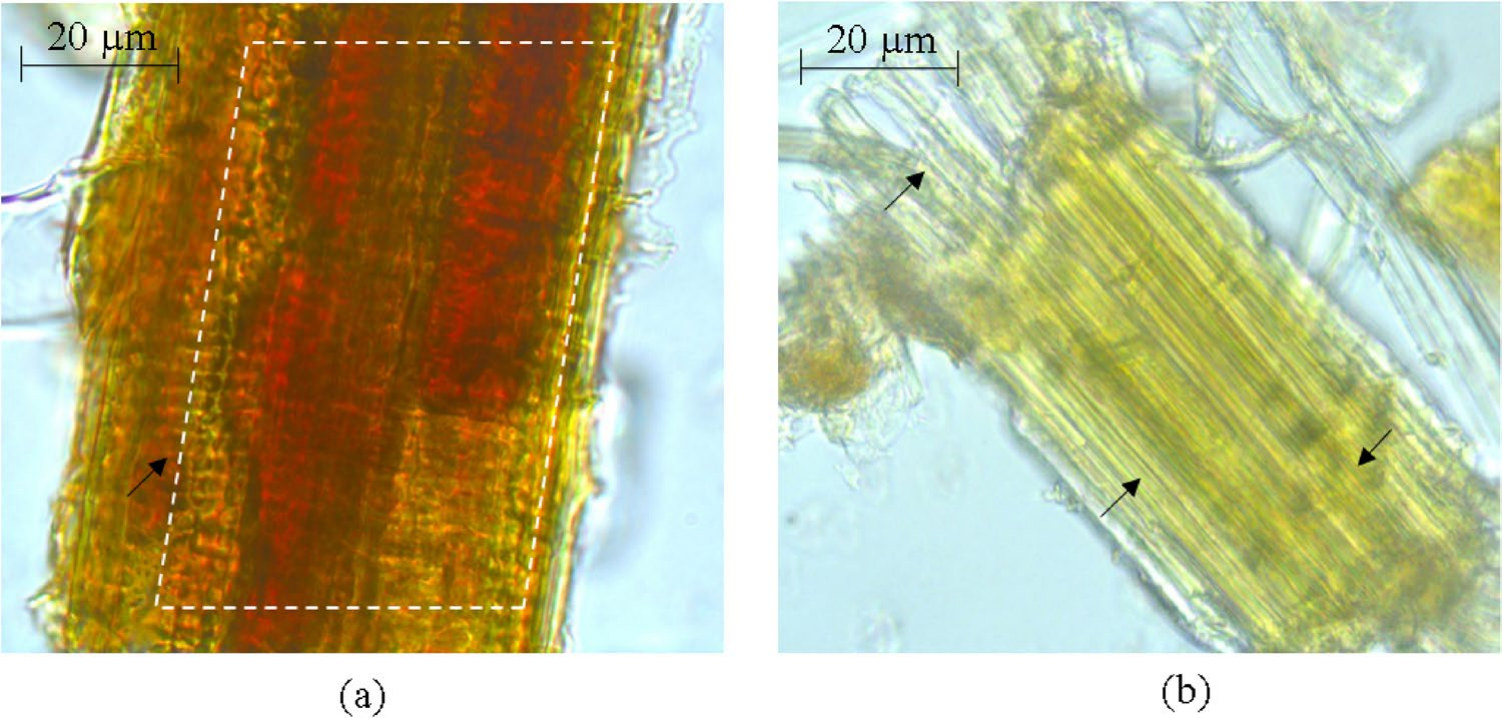
Bright field microscopy of untreated (**a**) and DES-THF pretreated (**b**) rice straw (magnification-×100).

**Figure 2:**
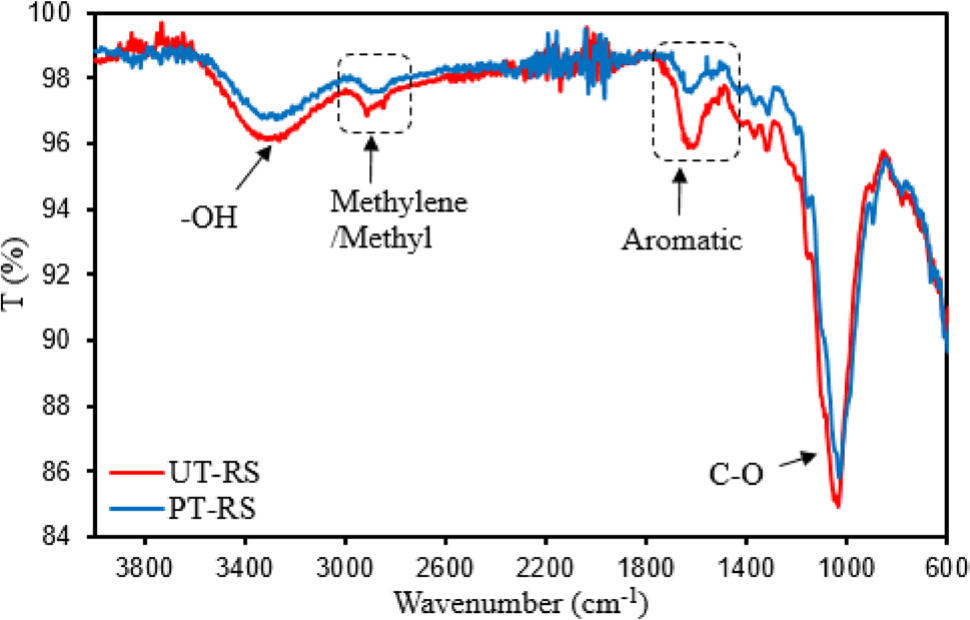
FTIR spectra of untreated (UT-RS) and DES-THF pretreated rice straw (PT-RS).

The changes occurring in rice straw upon pretreatment with DES-THF solvent system were determined by compositional, histochemical and spectroscopic (FTIR and EDX) analyses. The histochemical analysis of phloroglucinol (PG)-stained rice straw samples showed that untreated samples appeared dark brown (possibly due to the binding of phloglucinol with hydroxybenzaldehyde in lignin) (Fig. 1a), whereas the pretreated samples appear lighter or yellowish (probably due to the decreased lignin content and exposed cellulose microfibrils) (Fig. 1b). Microscopic observations of DE-THF treated and untreated samples matched with the results of compositional analysis which indicated that the pretreated rice straw samples had lower lignin content (~ 46%) and increased glucan content (~ 36%) over 10 solvent recycles (Table 1). The analysis of DE-THF treated solids by FTIR showed intensification of the corresponding peaks (1031 cm^−1^ and 3400 cm^−1^) due to C–O stretching and O–H bending^38,39^ (Fig. 2), thereby indicating no changes in cellulose and hemicellulose concentrations. As expected, the peak intensities at 1549 cm^−1^, 1644 cm^−1^ (due to C=O stretching of aromatic ketones in lignin) and 2885 cm^−1^ (related to C–H stretching of lignin) had reduced (Fig. 2) due to the reduced lignin content^39^.

**Table 1:** Composition analysis of untreated (UT-RS) and pretreated rice straw (PT-RS) samples during subsequent solvent reuses (C1–C10); all of the percentage compositions of raw and pretreated solid samples were calculated based on their dry weights.

Sample	Glucan (%)	Xylan (%)	Arabinan (%)	Lignin (%)	Ash (%)	Ext. (%)
UT-RS	31.4 ± 0.5	19.9 ± 0.8	2.7 ± 0.2	19.1 ± 0.7	11.4 ± 1.5	12.5 ± 1.2
PT-RS C-1 solids	41.1 ± 0.7	18.1 ± 0.7	2.9 ± 0.0	10.5 ± 0.1	14.8 ± 1.2	ND
PT-RS C-2 solids	41.9 ± 0.6	18.4 ± 0.0	2.6 ± 0.0	10.17 ± 0.3	14.3 ± 1.8	
PT-RS C-3 solids	41.9 ± 0.2	18.4 ± 0.1	2.8 ± 0.0	10.33 ± 0.2	15.0 ± 1.1	
PT-RS C-4 solids	44.1 ± 0.2	19.8 ± 0.2	2.3 ± 0.5	10.0 ± 0.5	10.0 ± 0.8	
PT-RS C-5 solids	46.0 ± 1.0	20.5 ± 0.1	3.0 ± 0.1	10.3 ± 0.1	12.3 ± 1.2	
PT-RS C-6 solids	43.6 ± 0.6	20.3 ± 3.1	2.5 ± 0.3	11 ± 0.6	8.3 ± 1.5	
PT-RS C-7 solids	44.2 ± 0.3	24.5 ± 0.4	3.3 ± 0.5	10.0 ± 0.4	14.0 ± 0.9	
PT-RS C-8 solids	43.9 ± 0.8	24.9 ± 0.7	3.3 ± 0.2	12.7 ± 0.8	13.0 ± 1.1	
PT-RS C-9 solids	40.6 ± 0.5	16.4 ± 0.9	1.85 ± 0.5	8.0 ± 1.1	13.0 ± 0.9	
PT-RS C-10 solids	41.2 ± 1.1	17.4 ± 0.5	2.3 ± 0.2	9.0 ± 0.5	13.2 ± 0.5	

*Ext*. extractives, *ND* not determined.

Rice straw contains several metals such as K^+^, Al^3+^, Mn^2+^, Fe^3+^, Cu^2+^, Zn^2+^ etc., which can interfere with the action of cellulases during hydrolysis^40^. To ascertain the effect of DES-THF pretreatment on the metal content of rice straw, elemental analysis of untreated and pretreated rice straw samples was carried out using ED-XRF. The results indicated that pretreatment of rice straw containing the elements S, K, Mg, P, Ca Si, O, C, Al caused a reduction in contents of S and K and the removal of Mg, P and Ca (Table 2). The relative concentrations of Si and O increased due to removal of lignin and the corresponding increase in the carbohydrate content after DE-THF pretreatment. Another evidence for the selective removal of lignin comes from the determination of the oxygen: carbon (O/C) ratios of separated lignin and treated /untreated biomass samples. Usually, the O/C ratios of lignin and biomass samples are 0.511 and 1.23, respectively^41^. During our study, we found that the O/C ratio of pretreated rice straw (1.26) was three times higher than the untreated biomass samples (0.42), due to reduced O and increased C contents possibly due to the selective removal of lignin.

**Table 2:** Energy dispersive X-ray analysis of untreated (UT-RS) and pretreated rice straw (PT-RS) samples.

		
Element	Untreated rice straw (UT-RS)		Pretreated rice straw (PT-RS)	
	Weight %	Atomic %	Weight %	Atomic %
C	59.77	71.13	38.8	48.02
O	25.16	22.48	49.13	45.63
Mg	0.48	0.28	–	–
Al	0.73	0.39	0.74	0.41
Si	3.17	1.61	10.88	5.76
P	0.91	0.42	–	–
S	0.50	0.22	0.25	0.11
Ca	1.07	0.38	–	–
K	6.13	2.24	0.20	0.08
O/C	0.42		1.26	

### Evaluation of DES recyclability and efficacy

The process cost can be substantially lowered by the reuse of catalysts if the final product quality remains unaffected. During our study, the DE-THF solvent mixture recovered after pretreatment was reused for upto 10 subsequent pretreatments with an average solvent recovery ranging from 74 to as much as 100% (Table 3). The average cellulose and hemicelluloses recoveries were consistent and remained higher ~ 91% and ~ 68%, respectively, even after 10 recycles (Table 3). Similarly, the concentration of lignin removed from biomass (~ 46%) remained comparable during solvent reuse while the glucan concentrations varied only marginally (± 1.75) (Table 3). These minor variations in concentrations of different components (Table 3) could also be attributed to handling errors. Overall, our results indicate that the newly developed DES-THF system can be effectively used for biomass pretreatment under milder conditions and recycled multiple times without generating any additional by-products.

**Table 3:** Effect of recycling DES-THF solvent system on delignification, cellulose and hemicellulose recoveries during rice straw pretreatment (values presented are mean for n = 2 analyses).

Cycle no.	Volume of fresh ^a^DES-THF mix (mL)	Recycled DES-THF (mL)	DES Recovery (%)	Solid recovery (%)	Cellulose recovery (%)	Hemicellulose recovery (%)	Delignification efficiency (%)
Control-A	380 (only DES)			82.7	95.3	70.1	32.9
Control-B	380 (only THF)			93	98.5	93.6	2.6
C-1	380		74	72	94.4	67.3	44.7
C-2	100	280	79	69	91.5	64.6	46.5
C-3	80	300	79	68	91.0	64.0	45.6
C-4	80	300	79	64	89.2	62.4	47.4
C-5	70	310	82	64	93.8	66.9	45.8
C-6	60	320	84	69	95.9	70.0	42.1
C-7	50	330	87	67	93.9	86.9	47.4
C-8	40	340	89	65	91.4	81.9	33.2
C-9	20	360	95	66	85.7	53.9	57.9
C-10	0	380	100	67	87.8	58.6	52.6
^b^AVG-10			85	67	91.5	67.6	46.3

BDW (bone dry weight) of raw rice straw used in each cycle of pretreatment was 55 g (solids concentration ~ 15% *w/v*).

^a^The solvent mentioned above contains six parts of original DES and four parts of THF solvent.

^b^AVG-10: average recoveries of DE-THF solvent, treated solids, cellulose, hemicellulose and average delignification efficiency over ten subsequent pretreatment cycles.

### A mechanistic understanding of selective delignification by DES-THF system

**Figure 3:**
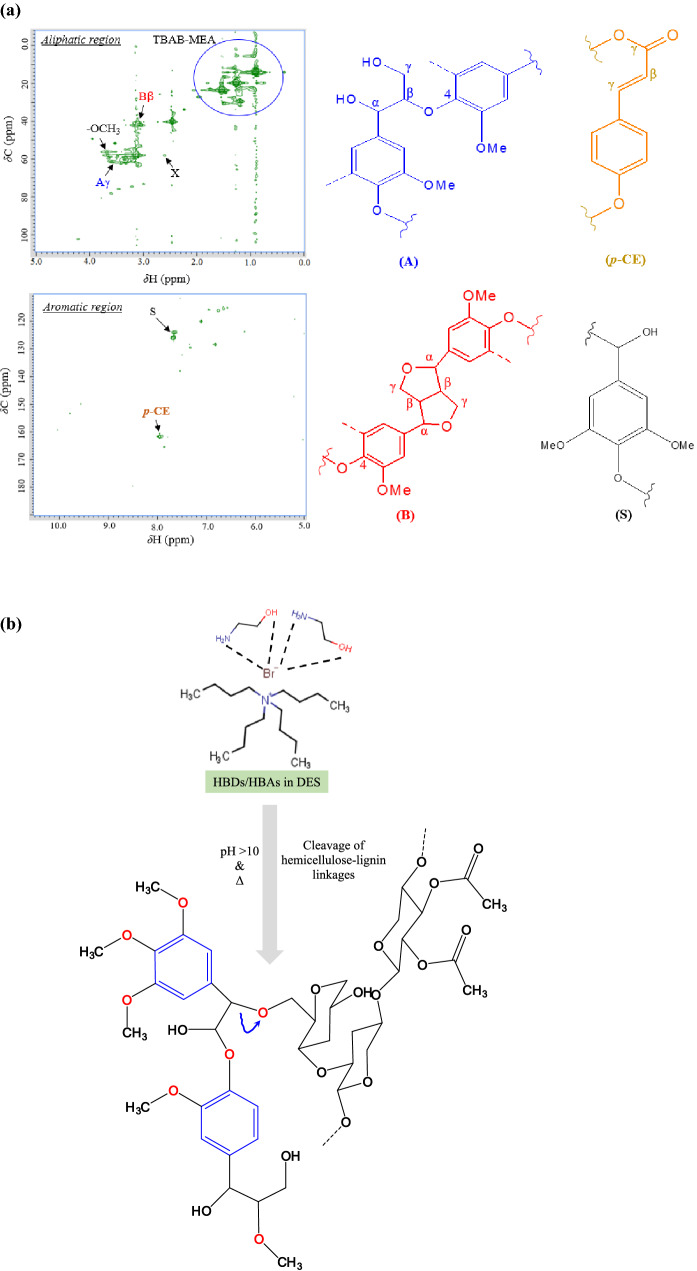
Analysis of lignin by (**a**) 2D-HSQC NMR indicating Cγ–Hγ of β-O-4 structures (Aγ), p-coumarate (p-CE) units, Cβ-Hβ of resinol structures (Bβ) & syringyl (S) units; (**b**) plausible mechanism of xylan and lignin cleavage catalyzed by DES solvent system.

In order to understand the plausible chemistry behind the selective extraction of lignin from rice straw by the newly developed solvent system, we first analyzed the structural features of isolated lignin samples using a 2D-HSQC NMR. The NMR spectrum of isolated lignin is presented in Fig. 3a, with corresponding assignments of monolignol units and lignin-carbohydrate complexes. All signals were assigned according to the literature (see the Supplementary Table S1). ^1^H–^13^C HSQC spectrum of lignin sample may be divided into two regions: the side-chain/aliphatic (^13^C/^1^H 50–110/2.5–6.0 ppm) and the aromatic (^13^C/^1^H 100–150/5.5–8.5 ppm) regions. The spectrum showed prominent signals in the side-chain regions for Cγ-Hγ of β-O-4 structures (Aγ), Cβ-Hβ of resinol structures (Bβ) and methoxyls (–CH_3_) as reported earlier^42,43^. Low-intensity signals corresponding to β-d-xylopyranoside units were also observed in the aliphatic region^42,43^ which indicated that hemicellulose was retained in the pretreated solid fraction. The signals in the aromatic region of spectra were related to the syringyl (S) and *p*-coumarate (*p*-CE) units. Overall, the results from ^1^H–^13^C HSQC NMR analyses revealed the presence of ‘S’ as a major lignin component in THF-DES extracted rice straw lignin.

**Figure 4:**
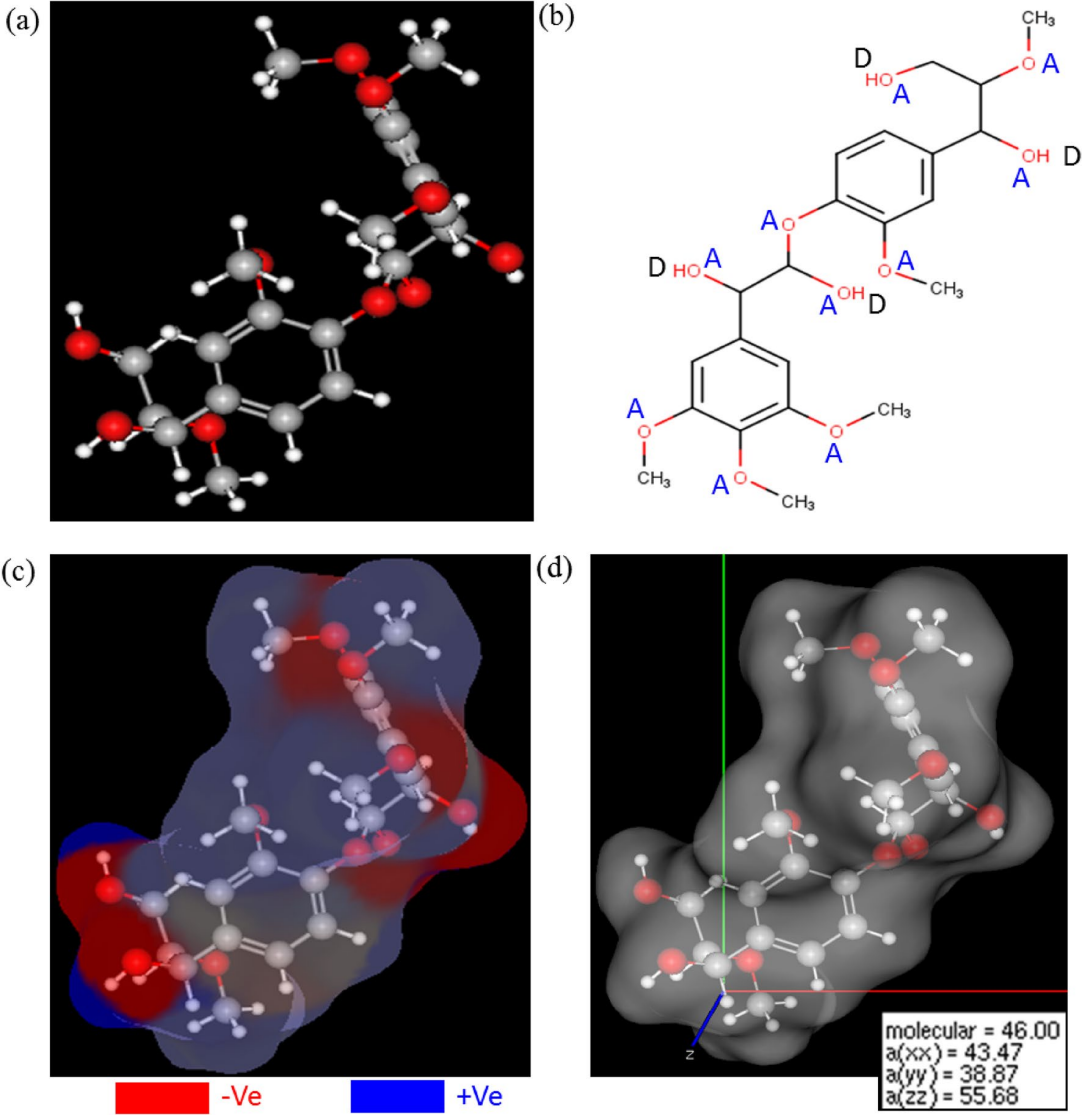
Model unit of S-lignin (1‐{4‐[1,2‐dihydroxy‐2‐(3,4,5‐trimethoxyphenyl)ethoxy]‐3‐methoxyphenyl}‐2‐methoxypropane‐1,3‐diol) (**a**); the figure shows the presence of hydrogen bond acceptors and donors (**b**), the charge distribution (**c**) and molecular polarizabilities (**d**) on s-lignin unit.

Based on the above structural features of isolated lignin, a model S-lignin unit ((1‐{4‐[1,2‐dihydroxy‐2‐(3,4,5‐trimethoxyphenyl)ethoxy]‐3‐methoxyphenyl}‐2‐methoxypropane‐1,3‐diol)) was built using MarvinSketch (version 2019)^44^ and was analyzed for various molecular properties viz*.* the presence of hydrogen bond acceptors (HBAs)/donors (HBDs), the electrostatic mapping and molecular dipolarity/polarizability (Fig. 4). The model lignin unit contained about ten HBA sites and four HBD sites (Fig. 4b) and its electrostatic potential map was displayed in Fig. 4c (for charge values see Supplementary Fig. S1). The mean polarizability of model unit was 46 C m^2^ V^−1^ (the average over the x, y and z axes of the molecule) which influences the overall reactivity of lignin (Fig. 4d). Typically, the polarizability/dipolarity (π*) of a compound indicates the ability of that compound to form instantaneous dipoles which is essential for its dissolution in the employed ionized solvents. Our results showed that the model lignin S-unit has considerable inherent dipolarity due to which it can interact with components of the present DE solvent.

Based on the results obtained from lignin analyses, a probable mechanism of lignin extraction is presented in Fig. 3b. Lignin is a complex aromatic biopolymer that together with hemicelluloses strengthens the plant secondary cell walls and both the natural components are held together mainly by the aryl ether or ester bonds in addition to extensive hydrogen bonding^45^. Under alkaline conditions, almost all of the lignin gets solubilized^46,47^ but even the hemicelluloses are lost. The solvent mixture developed in our study is alkaline (pH 10–12) and therefore provides a suitable environment for selective lignin dissolution with minimal loss of hemicelluloses. Moreover, the presence of THF in the mixture reduces the overall viscosity of the DES (Table 4) that may promote stronger solute diffusivity within the biomass^48^, necessary for effective lignin removal. In addition, THF can also dissolve hydrophobic regions leading to selective lignin displacement^49–51^. We further envisage that the use of DES-THF mixture may weaken the hydrogen bonds in lignin–hemicellulose complex, thereby causing its displacement. In addition, the presence of bromide, a strong hydrogen bond acceptor presents in DES and ethanolamine may respectively form bonds with the donors/acceptors on hemicellulose or lignin and break the ether linkages Fig. 3b.

**Table 4:** Properties of DES system at ambient conditions.

S. no.	Sample	Density (g/mL)	Viscosity (cP)	Conductivity (µS/cm)
1	THF	0.889	0.48	0.336
2	^a^DES	1.01	38.8	1456
3	DES-THF	0.911	5.3	2450

*THF* tetrahydrofuran.

^a^TBAB-MEA deep eutectic solvent.

### Amenability of pretreated biomass to cellulolytic enzymes

**Figure 5:**
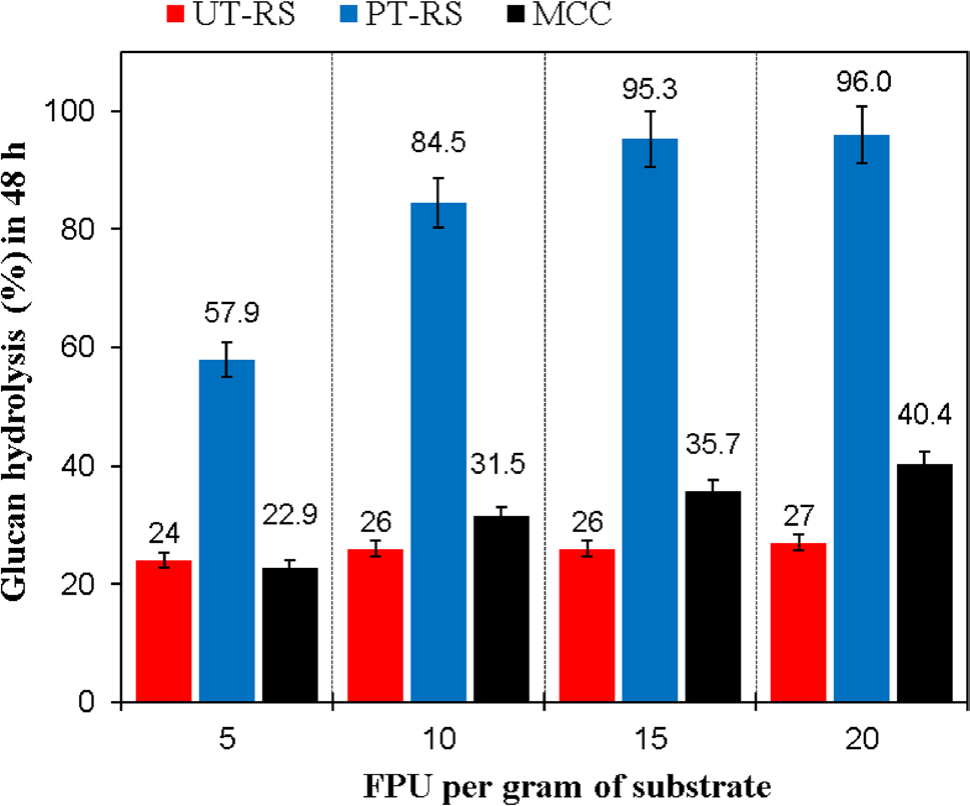
Enzymatic conversion of untreated (UT-RS) and pretreated rice straw (PT-RS) in comparison with MCC (control) at different cellulase dosages incubated at 50 °C for 48 h.

The effectiveness of the newly developed DES-THF system was established by the extent of lignin removal and by determining the ease of cellulose hydrolysability (Fig. 5). The enzymatic hydrolysis of DES-THF treated biomass showed that the percentage hydrolysis (~ 58%) obtained at lower enzyme dosage of 5 FPU g^−1^ was ~ twofold higher than that obtained with untreated rice straw and MCC (Fig. 5). Further increase in enzyme dosages to 10 FPUg^−1^ and 15 FPUg^−1^ resulted in rice straw hydrolysis of 84.5 and 95.3% respectively while on adding 20 FPUg^−1^ of enzyme, the improvement in hydrolysis (96%) was marginal. On the other hand, the percentage hydrolysis of UT rice straw and MCC at 20 FPU was around 40% and 27%, respectively (Fig. 5). The results indicated that DES-THF pretreated rice straw was highly amenable to lower enzyme dosages.

### Structural aspects of cellulose in pretreated biomass

In order to determine the reasons for obtaining highly amenable cellulose from DES-THF pretreated rice straw, detailed structural analysis w.r.t crystallinity index, crystallite sizes, and *d*-spacing was carried out using wide angle X-ray diffraction (WAXD) and solid-state NMR spectroscopy (^13^C cross polarization/magic angle spinning) techniques. Earlier studies have shown that native cellulose component in biomass exists as a semi-crystalline structure (cellulose-Iβ form) wherein parallel chains of the glucan polymer are juxtaposed together to form flat sheets bound by inter-hydrogen linkages^33^. Usually, all the known cellulose lattice planes (1–10, 110, 004, 021, 200) are observed in cellulose-Iβ form which is common in higher plants^52^ while commercially available microcrystalline cellulose (MCC) predominantly comprises of lattice plane 200 due to its alkaline/acidic synthesis method^53^. The cellulose lattice planes 1–10, 110 and 004 are relatively less abundant than other planes.

**Figure 6:**
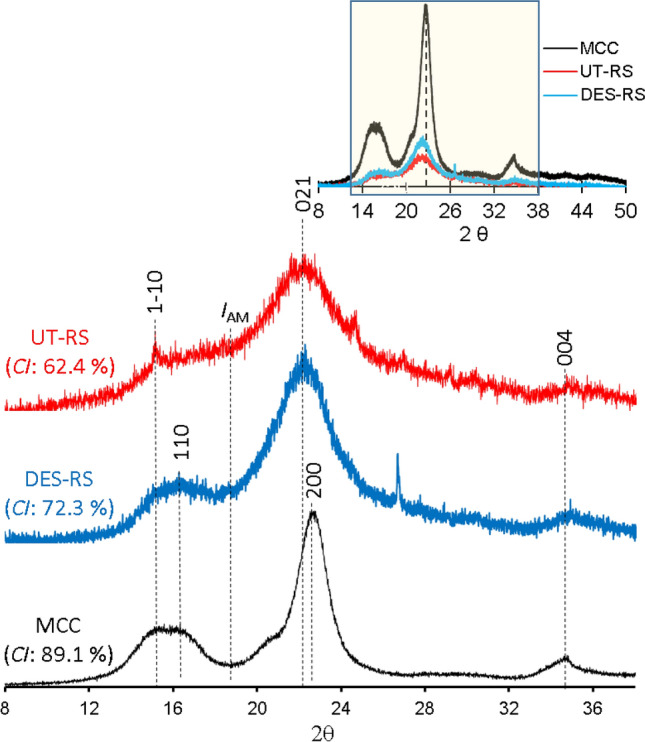
XRD patterns of untreated (UT-RS) and pretreated rice straw (DES-RS) in comparison with microcrystalline cellulose (MCC).

Figure 6 shows the X-ray diffraction patterns of untreated (UT) and pretreated (PT) samples in comparison with microcrystalline cellulose (MCC), chosen as the reference compound. Usually, the cellulosic fraction in untreated rice straw samples would be of the Iβ type consisting of all the planes; however, our analysis did not detect plane 110, probably due to masking by lignin. On delignification by DES-THF, the plane 110 was revealed, while the lattice planes 200 and 1–10 were not detected. On analysis of the crystalline planes of MCC sample, plane 200 was detected and 021 was not observed. The differences in detection of different crystalline lattice planes in rice straw (pretreated/untreated) and MCC samples could be attributed to the differences in the hydrogen-bond intensity (orderliness) between the adjacent cellulose chains in those samples. In order to determine the lattice planar composition of DES-THF pretreated rice straw, areas corresponding to individual XRD peaks were estimated by the peak deconvolution method (Lorentz fit) (Supplementary Figs. S2, S3). While both, UT and PT samples showed cellulose planes 021 and 004, the percentage of 021 lattice plane increased from ~ 54.6% (UT) to ~ 69.5% in PT sample, while the values for 004 decreased from ~ 26.0% (UT) to ~ 17.8% in PT biomass sample. The plane of 1–10 (~ 19.5%) in UT was not detected in PT samples possibly due to structural alterations whereas the undetected plane of 110 was observed only in PT sample (~ 12.7%), possibly due to delignification. The plane of 200 was not detected in both, UT and PT samples. Meanwhile in MCC sample, the plane of 200 (due to highly organized cellulose chains) was prominent (~ 57.6%) than other planes (021 1–10, 110 or 004).

Further analysis of the 2θ angles that coincide with the corresponding crystalline planes indicated that for UT and PT samples, the 021 plane (corresponding to 22.1°) remained unchanged. On the other hand, for PT rice straw samples, the diffraction observed at angle 15.4° (corresponding to plane 1–10 of UT samples) was not observed while that at 16.2° (corresponding to plane 110) was detected and is in line with an earlier report^54^. Similarly, for MCC, the predominant crystalline plane of 200 corresponded with 2θ of 22.7°, as reported earlier^55,56^. This clearly indicated that the orientation of the major crystalline plane (021) remained unaltered during DES-THF pretreatment. The angle of diffraction is mainly influenced by the changes in the alignment of the glucan chains constituting the cellulose structure.

### Crystallinity index and crystallite size

The relative fraction of the crystalline component in the cellulose is referred to as the crystallinity index (*CrI*). The crystallinity index of UT and PT samples was calculated by the Segal empirical equation^57^ and compared with the values obtained for MCC. For UT rice straw, the *CrI* was the lowest (62.4%) and upon pretreatment with DES-THF, increased to 72.3%. For MCC, the *CrI* value obtained (89%) was in line with earlier reports^58,59^ while the lower crystallinity of untreated rice straw samples could be due to the presence of lignin and hemicelluloses, their delignification by DES-THF may have increased the crystallinity index of pretreated solids.

**Figure 7:**
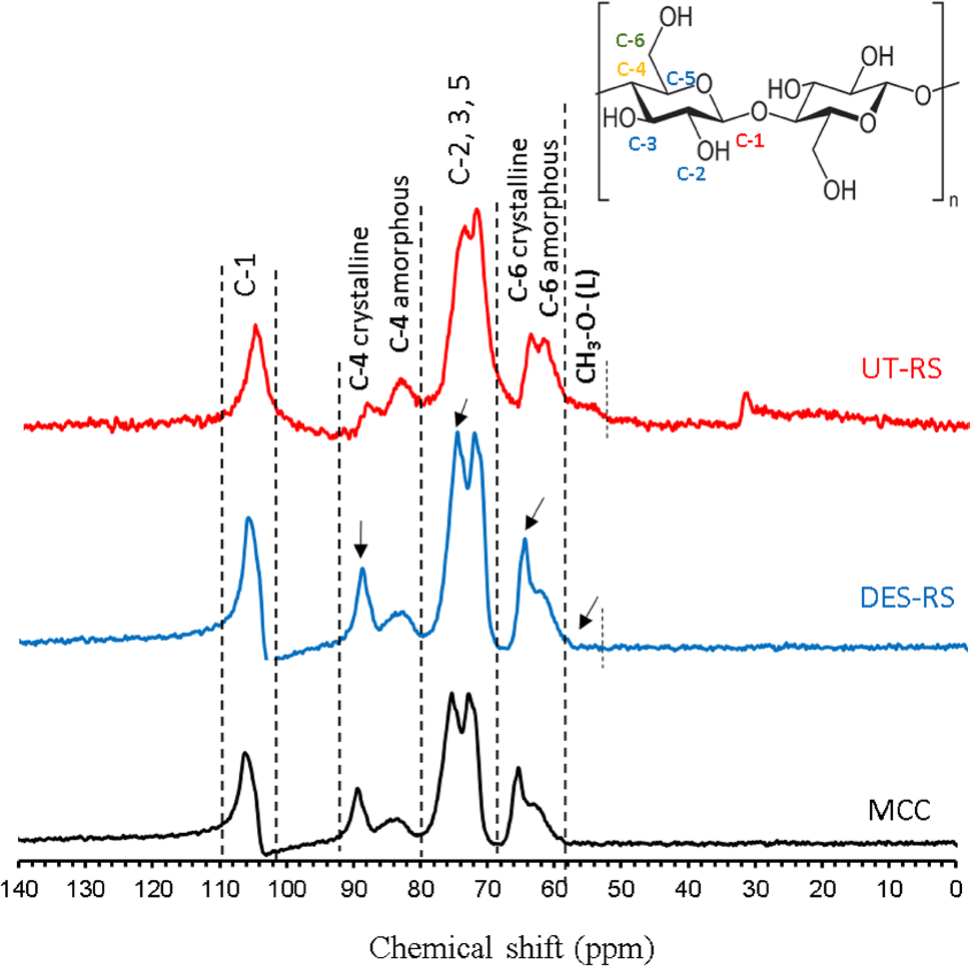
Solid state ^13^C CP/MAS NMR spectra of untreated (UT-RS) and pretreated rice straw (DES-RS) in comparison with microcrystalline cellulose (MCC); the arrows show the changes in the cellulose after pretreatment.

Additionally, we also determined the change that occurred in crystalline and amorphous regions of cellulose in rice straw during DES-THF pretreatment though CP/MAS ^13^C-NMR analysis (Fig. 7). All the peak assignments for samples were done based upon earlier reported chemical shift values as well as the shifts obtained for standard crystalline cellulose that was employed in our study. Broadly, the region between 60 and 110 ppm represents the polysaccharide fraction (cellulose and hemicellulose), while the chemical shift in the region of 55–60 ppm represents the methoxyl groups of lignin whose intensity was reduced in the pretreated sample owing to decrease in the lignin content (Fig. 7). Further, the intensities of C4 and C6 of crystalline cellulose components increased in the pretreated sample and this observation was in agreement with the results of crystallinity index as determined by XRD (Fig. 6).

Although several studies have directly linked higher crystallinity with increased cellulose recalcitrance and lower cellulase accessibility^60–62^, during our study, we observed that although the crystallinity of PT samples was only 18.7% lower than MCC, its hydrolysis by cellulases at 5, 10 and 15 FPU was ~ 2.5 folds higher than MCC. A better understanding of this observation would come after comparing our results with a recent report where cellulosic substrates were treated with TFA (trifluoroacetic acid) and phosphoric acid and converted into their amorphous forms^63,64^. After TFA treatment, the cellulosic substrates, namely Sigmacell and Avicel, were transformed into their amorphous forms with corresponding *CrI*’s of 42.1% and 37.6%^63^. Likewise, phosphoric acid pretreatment of the two cellulose substrates resulted in the formation of amorphous substrates with *CrI*’s of 25.3% (for Sigmacell) and 29% (for Avicel)^64^. These amorphous forms were then hydrolysed by 7 FPU of CTec2 (Novozymes) to obtain 85–90% hydrolysis^64^. On the other hand, during our studies, the DES-THF pretreated substrate with a *CrI* of 72.3% was easily converted at 5, 10, 15 and 20 FPU of cellulases to obtain hydrolysis of 57.9% w/v, 84.5%, 95.3% and 96.0%, respectively. The reasons for higher hydrolysis could be attributed to selective removal of lignin without affecting other components (hemicellulose and cellulose) during pretreatment.

In order to gain further insights into the structure of PT-RS cellulose, crystallite sizes perpendicular to the different lattice planes (based on the XRD diffraction patterns) were determined and the changes occurring in the cellulose fibril dimensions were estimated (Table 5). As explained earlier, the cellulose planes 021 and 004 were observed in both, UT-RS and PT-RS, while the planes 110 and 1–10 were only detected in the PT-RS or UT-RS, respectively. The *Dhkl* and *d*-spacing values refer to the crystallite sizes and inter-fibrillary spacing, respectively^46^. In UT-RS, the crystallite size (*Dhkl*) and inter-fibrillary spacing (*d-*spacing) values for cellulose lattice planes 1–10, 021 and 004 were obtained since other planes (110 and 200) were absent. After pretreatment of rice straw, the *Dhkl* and d-spacing values for only planes 021 and 004 were observed and 1–10 was absent. It was observed that the *Dhkl* values of pretreated rice straw were increased whereas the *d*-spacing values got decreased. The *Dhkl* and *d* spacing values obtained for different planes in MCC are in line with earlier reports^53^. The removal of lignin and some amount of hemicelluloses probably caused coalescence of cellulose crystallites leading to increase in their sizes (*Dhkl*) despite the reduction in inter-fibrillary distances (*d*-spacing). Generally, both, lower *Dhkl* and *d* spacing values of celluloses would result in reduced cellulase accessibility^65^, however, during our study the DES-THF pretreatment of rice straw samples caused an increase in *Dhkl* despite the lowering of *d-*spacing values.

**Table 5:** Crystallite sizes, inter-planar (d) spacing, crystallinity indices of rice straw cellulose before and after DES pretreatment, with microcrystalline cellulose (MCC) as a reference.

Sample	*CI* %	Lattice planes									
		(1–10)		(110)		(021)		(200)		(004)	
		*D(hkl)* (nm)	*d*-spacing (nm)	*D(hkl)* (nm)	*d*-spacing (nm)	*D(hkl)* (nm)	*d*-spacing (nm)	*D(hkl)* (nm)	*d*-spacing (nm)	*D(hkl)* (nm)	*d*-spacing (nm)
UT-RS	62.4	4.40	0.0317	–	–	1.48	0.0949	–	–	1.65	0.0856
^a^PT-RS	72.3	–	–	2.25	0.0622	2.15	0.0656	–	–	2.63	0.0551
MCC	89.1	4.23	0.0330	4.68	0.0299	–	–	4.5	0.0314	3.99	0.0364

*UT-RS* untreated rice straw, *MCC* microcrystalline cellulose, *CI* crystallinity index.

^a^Pretreated rice straw.

### Molecular modeling and properties of cellulose crystal lattice planes

Earlier reports show that the supramolecular structure of cellulose influences its physical properties such as solubility, dielectric relaxation, dipole moment, etc. Among these, the dipole moment is a measure of the net polarity of a molecule which decides its solubility and therefore its reactivity, and usually varies for each of cellulose allomorphs.

**Figure 8:**
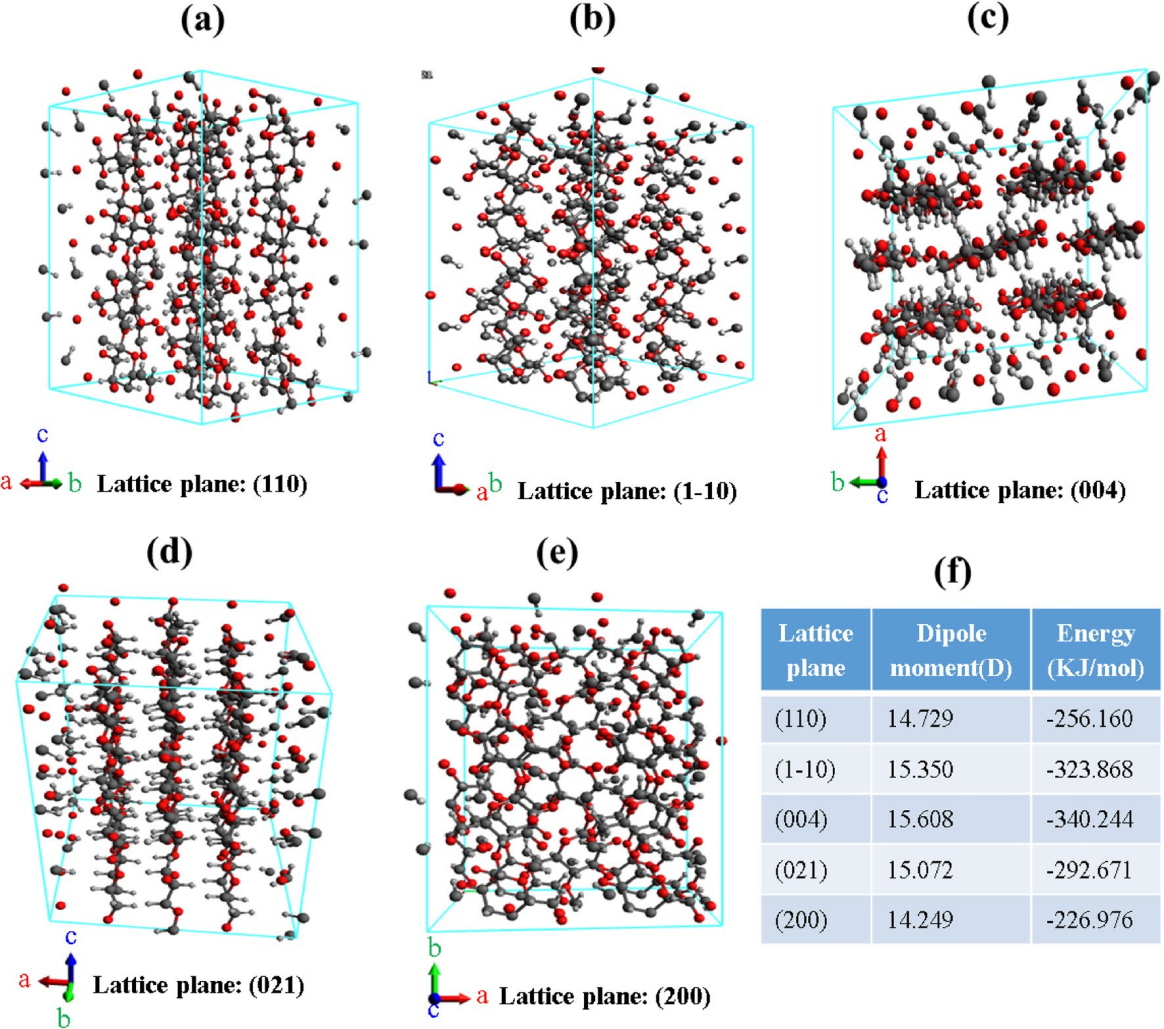
Different types of crystalline cellulose lattice planes and their estimated properties; the figure shows 2 × 2 × 2 super cell of cellulose crystal structure with lattice planes (110, 1–10, 004, 021 and 200); a total of 576 atoms and 493 bonds are present in each crystal structure.

In order to gain insights on the supramolecular properties of cellulose-Iβ allomorph, we designed a model cellulose crystal structure (2 × 2 × 2) using computational methods and determined various properties (viz. dipole moment, energy, number of bonds, type of bonds etc.) for all the different planes (Fig. 8). All the properties were measured at temperature of 300 K and the geometries of all these crystal planes were optimized under General Amber Force-field (GAFF) before estimating their properties. The results showed that the highest dipole moment of 15.608 dye was observed for 004 plane (amorphous) while the lowest of 14.249 dye was observed for 200 plane (more crystalline) (Fig. 8f). For lattice planes 110, 1–10 and 021 (with intermediate crystallinities), the corresponding dipole moments were 14.729, 15.350 and 15.072 dye. The energy content values determined appear to be directly proportional to the dipole moment, however, this needs detailed investigations.

## Conclusions

A newly developed solvent system comprising of DES and THF was successfully employed to carry out the selective delignification of rice straw for upto 10 recycles under milder pretreatment conditions. The consistent cellulose and hemicellulose recoveries obtained during subsequent reuse of the solvent system indicated high recyclability potential of the present DES based solvent system. Interestingly, despite an increase in crystallinity of cellulose after DES-THF pretreatment, the pretreated biomass could be easily hydrolyzed by cellulases at even lower dosages. Overall, our study indicates that reducing cellulose crystallinity during pretreatment may not be a prerequisite for effective enzymatic hydrolysis.

## Experimental

### Materials

Rice straw was procured from local sources around Bangalore city in India. The dried (< 5% moisture content) and size reduced biomass (10–15 mm) was used in the pretreatment experiments. All the chemicals required for the present study were of analytical grade and purchased from HiMedia (Mumbai, India). Cellulase enzyme (SachariSEB C6 Plus) used in this study was procured from Advanced Enzymes, Mumbai, India.

### Preparation of DES

Tetra-*n*-butylammonium bromide (TBAB) was mixed with 2-aminoethanol to obtain the DES. The binary mixture with a molar ratio of (1:2) was allowed to react under atmospheric pressure at 50 °C for about 2 h (Supplementary Scheme S1). Afterwards, the mixture was cooled down to room temperature and mixed with tetrahydrofuran. The final solvent system contained six parts (*v/v*) of DES and four parts (*v/v*) of tetrahydrofuran.

### DES-THF pretreatment and recyclability

All pretreatment studies were conducted in a 1 L batch reactor (Series. 4520, Parr Instruments, USA). About 55 g of raw rice straw was pretreated with 380 mL of DES-THF system at 100 °C for 3 h. After completion of reaction, the solid and liquid phases were separated by manual pressing and filtration. The recovered solids were washed with ~ 500 mL of ethanol to obtain residual DES trapped in the treated solids. The ethanolic wash was added to the lignin-rich hydrolysate and the whole mixture was kept at 4 °C for settling of precipitated lignin. After separation of lignin, the diluted DES-solvent mixture was subjected to rotary evaporation (Buchi Rotavapor R-215) for the separation of ethanol and THF from the original DES. Afterwards, DES was reconstituted from the recycled solvents and employed for next cycle of pretreatment with fresh rice straw material. The recycling procedure was performed for about ten successive pretreatment cycles and all the pretreated rice straw samples were analyzed for their chemical composition. The schematic diagram of the pretreatment process and the photographs of untreated, pretreated rice straw and isolated lignin are shown Supplementary Scheme S2 and Supplementary Fig. S4, respectively (Supplementary Information).

### Compositional analysis

The untreated and the pretreated rice straw samples (obtained from 10 cycles) were analyzed for their major constituents; viz*.* glucan, xylan, arabinan, lignin, ash and extractives (for untreated biomass) using the methods of National Renewable Energy Laboratory^66^. Briefly, 300 ± 1.0 mg of biomass sample (moisture < 10%) was pre-hydrolyzed with 3 mL of 72% (*w/v*) sulphuric acid for 1 h at 30 ± 2 °C in a Teflon screw-capped pressure tubes and after pre-hydrolysis, the acid concentration was reduced to 4% (*w/v*) by diluting with 84 mL of distilled water and subsequently autoclaved at 121 °C for 60 min. Afterwards, the hydrolysate was analyzed for monomeric sugars which were then used for determining the corresponding polymers based on the external sugar recovery standards. The solid residue obtained after acid digestion was used for the analysis of klason-lignin and ash through gravimetric analysis^66^.

### Enzymatic hydrolysis

Enzymatic hydrolysis experiments for untreated and pretreated rice straw samples were conducted in 100 mL screw capped Scott bottles at 50 °C in a shaking incubator at 150 rpm for 48 h. One gram of sample (5% *w/v*) was suspended into 20 mL of 50 mM tri-sodium citrate buffer (pH 4.8) with four different enzyme dosages (5, 10, 15 and 20 FPUg^−1^ of substrate). Microcrystalline cellulose was used as a control for glucan conversion. One unit of enzyme activity is defined as the amount of enzyme that produces 1 µmol reducing sugar per minute in the reaction mixture under specified conditions. The total cellulase activity (Filter paper units) of the stock enzyme was determined according to the standard method of IUPAC^67^. The percentage glucan conversion was quantified by determining the net conversion of glucan to glucose using the following equation;

$${\text{Enzymatic }}\,{\text{hydrolysis }}\left( {\text{\% }} \right) = \left[ {\frac{Total\, amount \,of \,glucose \times 0.9}{{Total \,amount \,of \,glucan }}} \right] \times 100.$$

## Characterization

### Chromatography analysis

All the sugar analyses in the present study were carried out using a UHPLC (Ultra High Performance/Pressure Liquid Chromatography) (Agilent 1290 Infinity) equipped with a Hiplex H column (Agilent) connected to RID (Refractive Index Detector). The sugar recovery standards of glucose, xylose and arabinose were analyzed using their respective sugar calibration curves. The conditions employed were, 0.005 M H_2_SO_4_, mobile phase (Filtered and degassed); 0.6 mL min^−1^, flow rate; 60 °C, column temperature; 55 °C, detector temperature.

### XRD analysis

X-ray powder diffraction patterns of untreated and pretreated rice straw samples were obtained by X’pert^3^ instrument (PANalytical, Netherlands). The X-ray source was Cu K-alpha, 0.15418 nm (Bragg–Brentano geometry) at a wavelength of 1.5406 nm. The samples were scanned over the angular range 8–50°, 2θ with a step size of 0.030°, smooth operation (0.302) and step time of 0.5 s. *CrI* (crystallinity index) was determined by means of the following Segal empirical equation^57^ via the height of the 200 lattice diffraction peak (*I*_*200*_, 2θ = 22.5°) and the minimum intensity of diffraction between the 200 and 110 peaks (*I*_*am*_, 2θ = 18°).

$$CrI\left( \% \right) \, = \, \left( {I_{200} - I_{am} } \right]/I_{200} \times { 1}00.$$

The cellulose crystallite sizes perpendicular to different lattice planes (D_*hkl*_) were calculated by Scherrer equation^68^.

$${\text{D}}hkl = \frac{k\lambda }{{d {\text{Cos}} \theta }},$$

where λ is the X-ray wavelength, k is the crystallite shape factor (0.9), D_*hkl*_ is the angular FWHM in radians of the (hkl) line plane profile, and θ is the scattering angle.

The d-spacing (inter-planar spacing) for different lattices were calculated from refined unit cell dimensions using Bragg’s equation

$${\text{n}}\lambda \, = {\text{ 2d}}_{hkl} {\text{Sin}}\theta .$$

### Spectroscopy studies

The effects of DE-THF solvent pretreatments on the rice straw chemical compositions were studied using FTIR, NMR and X-ray spectroscopies.

The FTIR spectra were recorded over the wavenumber range of 400–4000 cm^−1^ using a Perkin-Elmer (USA) instrument connected with a mercury-cadmium-telluride detector in attenuated total internal reflection mode.

Solid state NMR measurements were conducted by a ^13^C CP/MAS NMR spectrophotometer (Bruker Avance III400MHz) operating at 100.59 MHz for ^13^C using a Bruker double-resonance 4-mm MAS probe head at ambient temperature. The samples were packed in a 4 mm ZrO_2_ rotor attached with a Kel-F cap and spun at 8000 Hz. CP/MAS (cross-polarization magic angle spinning) ^13^C data were acquired with a Bruker CP pulse sequence under the following acquisitions: pulse delay 4 s, contact pulse 2000 ms, and 2 k to 4 k numbers of scans. For 2D HSQC NMR spectra, ~ 50 mg of THF-DES extracted lignin was dissolved in 0.5 mL of DMSO-*d*_*6*_. In order to understand the mechanism of selective delignification by the present DE solvent system, a preliminary structural analysis of isolated lignin was performed based on the 2D-HSQC counter intensities of various linkages.

Energy dispersive X-ray (EDX) spectra were collected from an attached energy-dispersive spectrometer fixed on the scanning electron microspore (JSM-7610F, JEOL, Japan).

### Microscopy studies

The distribution of lignin in untreated and pretreated rice straw was visualized by staining with phloroglucinol-HCl solution^69^ with bright filed microscopy.

### Cellulose and lignin models

All the cellulose crystal structural models were prepared using the earlier reported crystallographic information files^33^ and analyzed in Avogadro^70^ with × 86–64-bit Intel platform. MarvinSketch^44^ was used for building model lignin units and analysis.

## Supplementary Information

Supplementary Information.

## Data Availability

All data generated or analyzed during this study are included in this published article.
