# Supplementary material for: Generation of highly amenable cellulose-Iβ via selective delignification of rice straw using a reusable cyclic ether-assisted deep eutectic solvent system
Source: Sci Rep. 2021 Jan 15;11:1591. doi: 10.1038/s41598-020-80719-x (PMC7810886; doi:10.1038/s41598-020-80719-x)
Supplement: Supplementary file 1 — Supplementary Information. [file 41598_2020_80719_MOESM1_ESM.docx]

**Generation of highly amenable cellulose-Iβ *via* selective delignification of rice straw using a reusable cyclic ether-assisted deep eutectic solvent system**

Thulluri Chiranjeevi, B. Ravi & Harshad Ravindra Velankar^*^

*^1^Bioprocess Group, Hindustan Petroleum Corporation Limited, HP Green R&D Centre, KIADB Industrial Area, Tarabahalli, Devanagundi, Hoskote, Bengaluru 560067, India*

**Supplementary Data**

**Scheme.S1. Synthesis of TBAB-MEA deep eutectic solvent system**

**
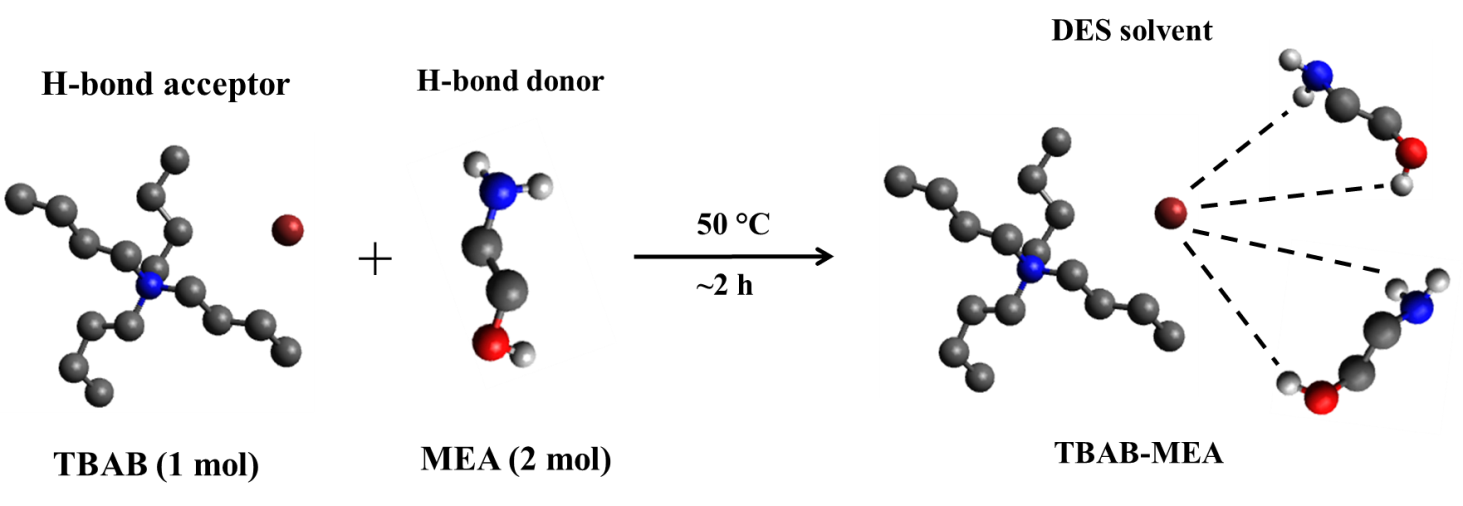
**

Tetra-n-butylammonium bromide (TBAB) was mixed with 2-aminoethanol to obtain the DES. The binary mixture with a molar ratio of (1:2) was allowed to react under atmospheric pressure at 50 °C until solid particles were disappeared (~2 h). Once the homogeneous and transparent liquid was formed, the mixture was cooled down to room temperature and used for pretreatment of rice straw in assistance with tetrahydrofuran (a final solvent system contains six parts of DES and four parts of THF).

**Scheme. S1. Process flow diagram of DE-(MEA: TBAB)-THF pretreatment of rice straw biomass**


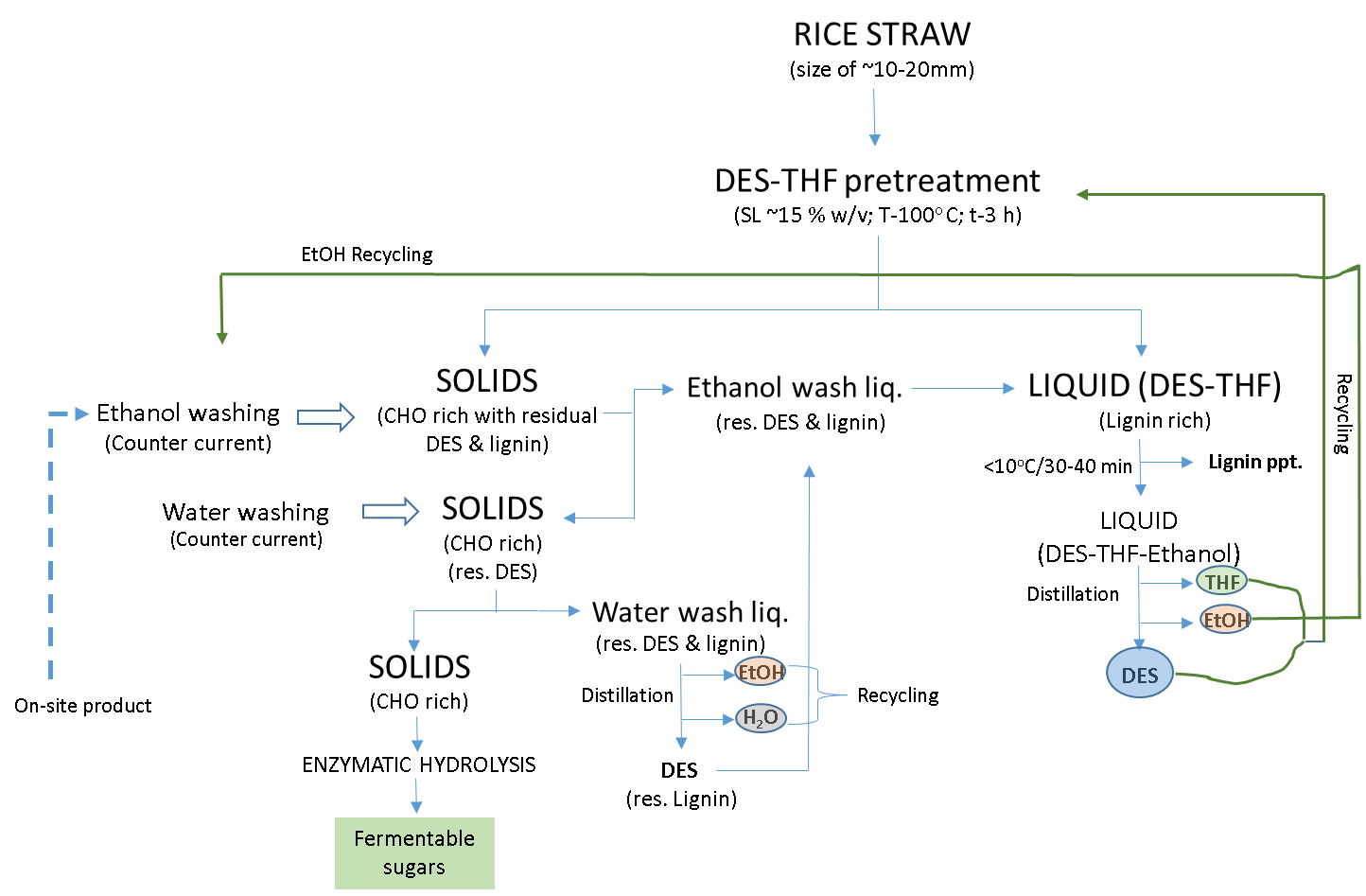


**Table. S1. Assignments of various signals obtained for DES-THF extracted rice straw-lignin sample in 2D HSQC-NMR spectrum**

| **Label** | ***δ_C_* /*δ_H_* (ppm)** | **Signal assignment** |
| --- | --- | --- |
| Aγ | 59.6−60.8/3.37−3.72 | Cγ−Hγ of β-O-4 structures |
| CH_3_O- | 55.5/3.76 | C-H in aromatic methoxyl groups |
| S | 100–150/5.5– 8.5 | Aromatic (S-lignin unit) |
| *p*-CE | 130/7.5 | *p*-coumarate |
| Bβ | 50/2.8-3.0 | Cβ-Hβ of resinol structures |
| X | 50-53/2.3 | β-D-xylopyranoside units |

**Fig.S1. Electrostatic potential map of model lignin unit (S-unit)**


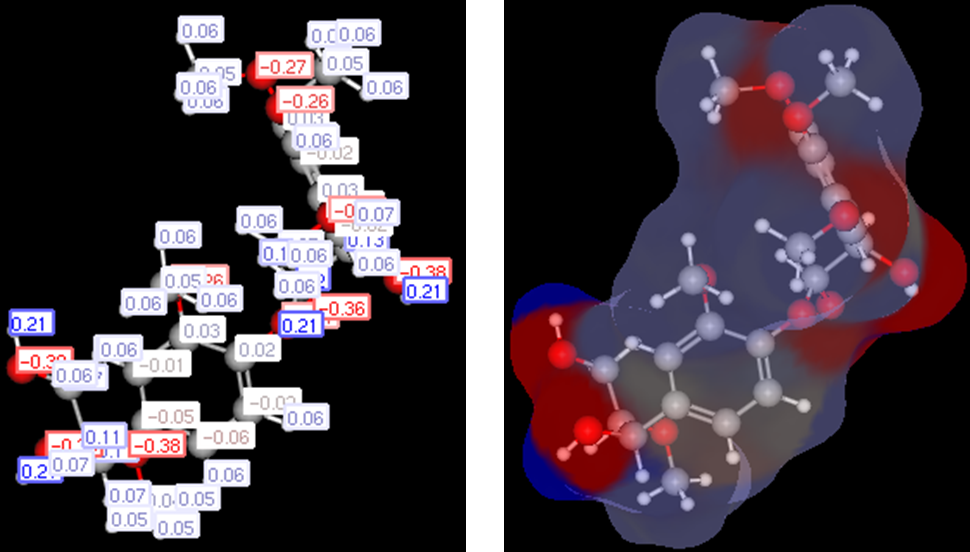


**Fig.S2. XRD-Peak deconvolution**


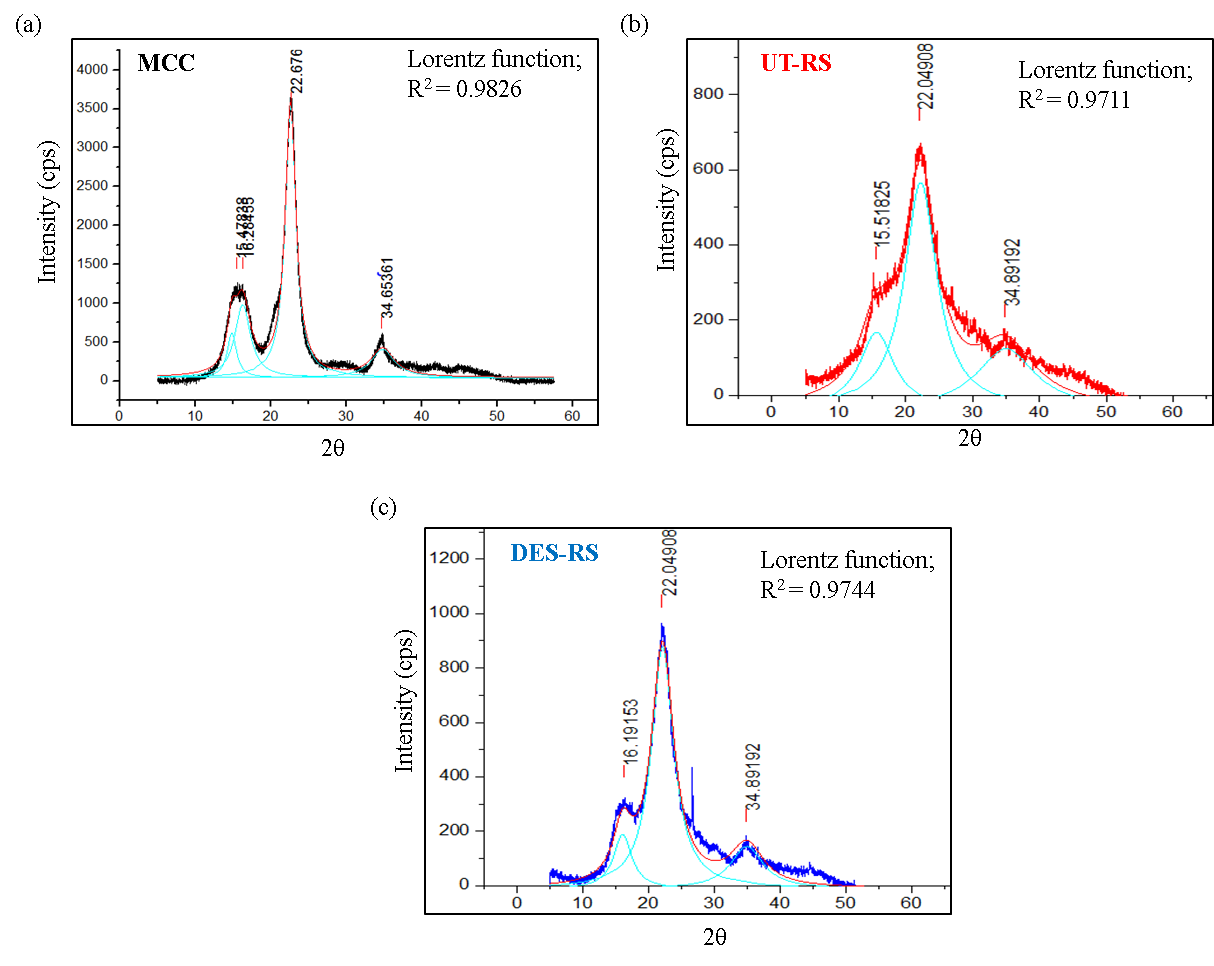


**Fig.S3. Types of cellulose lattice planes and magnitudes in microcrystalline cellulose (MCC), untreated and DES-THF pretreated rice straw celluloses**

**Fig.S4. Photographs of DES, DES-THF, raw rice straw, pretreated rice straw and isolated lignin from rice straw**


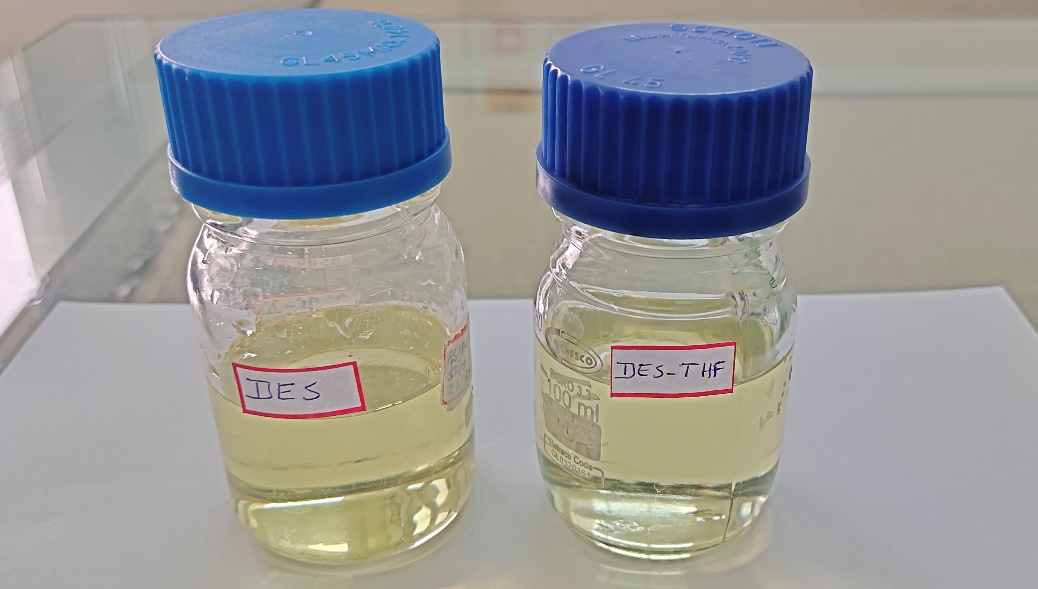


DES Solvent systems


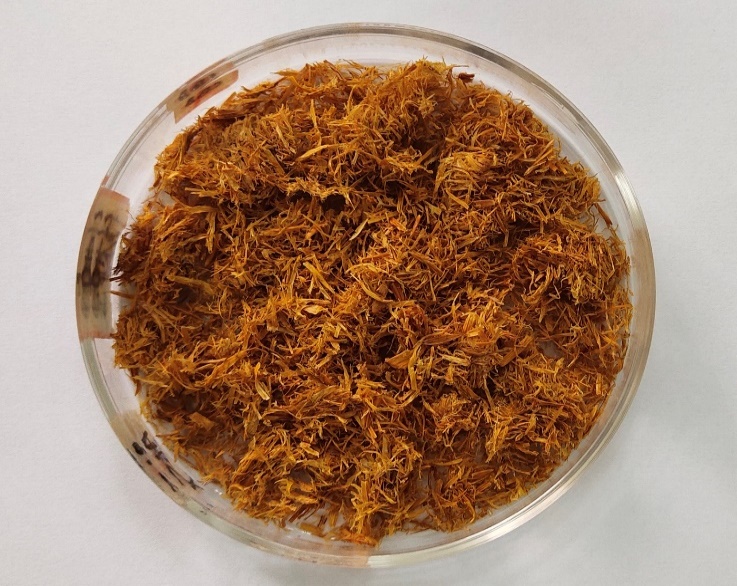

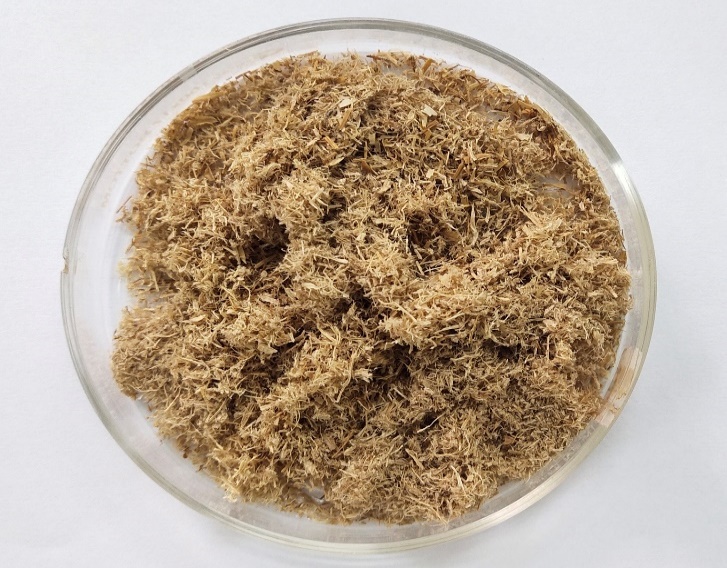


Untreated Rice straw

Pretreated Rice straw


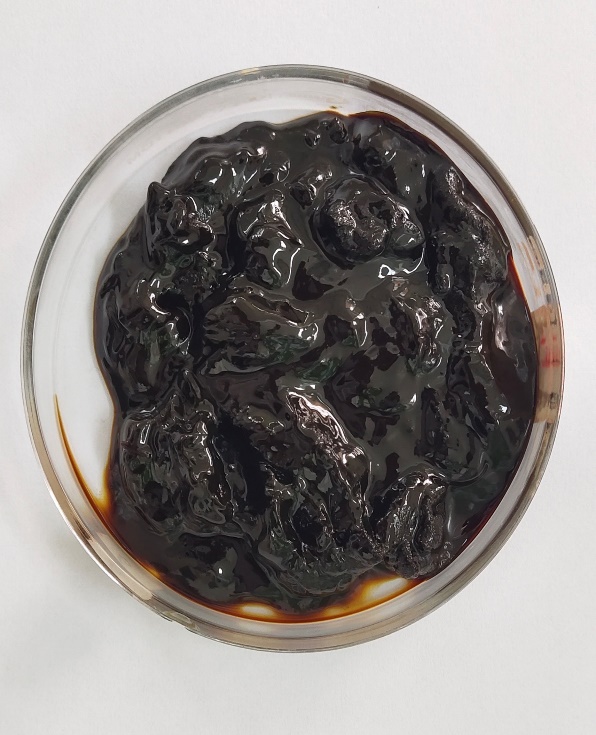


Isolated Lignin
